# Impact of COVID-19 vaccination on adolescent and youth students’ mental health and bullying behaviors after the lifting of COVID-19 restrictions in China

**DOI:** 10.3389/fpubh.2024.1469792

**Published:** 2024-12-16

**Authors:** Hong-Jun Song, Cong Wang, Yun-Fei Mu, Jia Cai, Zhongyue Deng, Yu Wang, Ai-Ping Deng, Ting Liu, Bin Li, Yi Huang, Jin Chen, Yan Hu, Bo Liu, Wei Zhang, Lin Lu, Mao-Sheng Ran

**Affiliations:** ^1^Mental Health Center, West China Hospital, Sichuan University, Chengdu, Sichuan, China; ^2^West China School of Nursing, Sichuan University, Chengdu, Sichuan, China; ^3^Institute of Psychiatry, West China Hospital, Sichuan University, Chengdu, Sichuan, China; ^4^Department of Clinical Epidemiology and Evidence-Based Medicine, West China Hospital, Sichuan University, Chengdu, Sichuan, China; ^5^Sichuan Second Veterans Hospital, Sichuan, China; ^6^Jingzhou Mental Health Center, Hubei, China; ^7^Peking University Sixth Hospital, Peking University Institute of Mental Health, NHC, Beijing, China; ^8^Key Laboratory of Mental Health, Peking University, Beijing, China

**Keywords:** COVID-19 vaccination, school bullying, mental health, adolescent, China

## Abstract

**Background:**

Vaccination plays an important role in fighting against COVID-19. However, it is unclear about the association among vaccination, mental health, and bullying behaviors in China.

**Method:**

This online survey was conducted to investigate the association among vaccination status, mental health problems and bullying behaviors in students from December 14, 2022 to February 28, 2023 in Sichuan, China. All participants (*N* = 82,873) were adolescents recruited via their teachers and professors. Patient Health Questionnaire-9 (PHQ-9), Generalized Anxiety Disorder-7 (GAD-7), PTSD Checklist for DSM-5 (PCL-5), Sleep Severity Index Scale (ISI) and Cyberbullying behaviors were tested.

**Results:**

The rates of depression, anxiety and PTSD in participants without vaccination were significantly higher than that in those with vaccination. Moreover, participants with more doses of vaccines had significantly lower rates of depression, anxiety, PTSD, school and cyber bullying (*p* < 0.001). The rates of homosexual orientation, drinking, smoking were higher in participants with three or more doses of vaccines than those with less doses of vaccines (*p* < 0.001).

**Conclusion:**

This study suggests that COVID-19 vaccination will not only protect students’ physical health, but also improve mental health. It is crucial to explore the mechanism between vaccination and mental health problems and bullying behaviors in further studies.

## 1 Introduction

Vaccination plays an important role in fighting against COVID-19, and evidence shows that vaccination provids a good protection for people in all over the world (1–3), especially in reducing severe cases, hospitalization, and death (4–6). Moreover, vaccination is more cost-effective than other public health policies and measures during the COVID-19 pandemic (4). The promotion of the vaccinations allowed public places such as schools and workplaces to reopen, and people’s daily lives are gradually back to normal (7). Given the importance of adolescent and youth students, the impact of vaccinations on these students should be further explored.

Previous studies have demonstrated that there is a correlation between vaccination and mental health during COVID-19 pandemic (8, 9). On the one hand, COVID-19 vaccination might significantly decrease anxiety (10, 11), depression (12, 13), insomnia (8), and posttraumatic stress disorder (PTSD) (14). On the other hand, COVID-19 vaccine hesitancy would contribute to deteriorated mental health status such as higher levels of depression and anxiety (14, 15). However, most previous studies focused on other population, but not students. Further studies should be conducted to explore the association between COVID-19 vaccination and mental health problems among adolescent and youth students.

Bullying is a repetitive, intentionally aggressive behavior, indicating an imbalance of power between the victim and perpetrator (16). Traditional bullying such as school bullying and workplace bullying, and cyberbullying have caught researchers’ attention (17, 18).Previous studies have shown that cyberbullying has increased during COVID-19 pandemic (17, 19), probably resulting from the increase use of internet (20). For traditional bullying, there is a sharp decrease of school bullying during COVID-19 pandemic (21, 22), indicating lower level of interaction among students at schools. The use of biosecurity measures such as wearing personal protective equipment may result in bullying (23). However, few studies have explored the possible relationship between vaccination and bullying during and after COVID-19 pandemic. Further studies should be conducted to identify the association between vaccination and bullying, especially after the lifting of COVID-19 restrictions in China.

This study aimed to explore the association between vaccination and mental health problems, as well as bullying behaviors among students from middle and high schools, colleges and universities in Sichuan, China.

## 2 Method

### 2.1 Design and participants

After the lifting of COVID-19 restrictions in China, the cross-sectional survey was conducted from December 14, 2022 to February 28, 2023 among students from middle and high schools, colleges and universities in Sichuan province, China. This study was approved by the Biomedical Research Ethics Committee of West China Hospital, Sichuan University (No: 2022–1790).

To ensure the quality of the research, the questionnaire information was first sent to teachers and professors at middle and high schools, colleges and universities in Sichuan Province, China. Then, this questionnaire information was directly disseminated by the teachers and professors to their students. Participation in the survey was entirely voluntary for students. Neither teachers, professors, nor school administrators could directly access students’ filled information, including whether a student participated in the survey, to minimize potential biases in the educational setting. Moreover, the confidentiality of all data was ensured. Questionnaires were completed by participants after they provided informed consent.

### 2.2 Measurements

This survey included four parts: the first part was demographic information, including gender, age, grade, sexual orientation, monthly family income, parents’ education level, parenting style and so on. The second part was information related to COVID-19 pandemic, such as the COVID-19 infection status, vaccination and willingness of vaccination, quarantine experience, and psychological stress. The third part was the impact of the COVID-9 pandemic on personal behavior habits, mainly including daily life routine, smoking, drinking, physical exercise, and bullying. The final part was the mental health problems, including depression, anxiety, PTSD, insomnia, and suicidal ideation.

The Patient Health Questionnaire (PHQ-9) is commonly used to measure depression symptoms in the past 2 weeks (24). There are 9 items with a total score of 0–27, and 5 points or above for depression symptom (25). The Generalized Anxiety Disorder-7 (GAD-7) is commonly used to assess degree of anxiety symptoms (26). The scale consists of 7 items with score ranging from 0 to 21. In this study, 5 was took as a cut-off point for anxiety symptom. Both PHQ-9 and GAD-7 had good reliability and validity in Chinese young people (24, 26).

The PTSD symptoms were measured using the PTSD Checklist for DSM-5 (PCL-5). The PCL-5 is a 20-item self-report assessment tool to measure the symptoms of PTSD based on DSM-5 diagnostic criteria, with a sum score of ≥33 suggesting symptoms of PTSD (27). Symptoms of insomnia was measured by the Sleep Severity Index Scale (ISI) (28). In this study, scores of >8 on the ISI (ranging from 0 to 28) were as cut-off points for identifying individuals with symptoms of insomnia, The Chinese version of PCL-5 and ISI had good reliability and validity (29).

School bullying was assessed by the 6-item scale from PISA (Program for International Student Assessment). The frequency of bullying was divided into four levels: “no or almost no,” “several times a year,” “several times a month,” and “once a week or more.” Any options were defined as school bullying victimization except “no or almost no” in this study. The Chinese Cyberbullying Intervention Project Questionnaire (C-CIPQ) consisting 14 items was used to measure cyberbullying behavior. Cyber bullying victimization and perpetration were scored from 0 to 21 respectively, and 1 was applied as a cut-off point in this study. The scale had a good convergent validity and discriminant validity (30).

### 2.3 Statistical analysis

According to the vaccination status and the times of dose, participants were divided into four groups. Participants who did not receive COVID-19 vaccine were included in Group 1 (G1), and participants who received one dose, two doses, three or more doses of vaccine were included in Group 2 (G2), Group 3 (G3), and Group 4 (G4), respectively. Descriptive analysis was used to analyze general information, ANOVA and *post hoc* comparisons were used to analyze differences between the four groups. The multiple logistic regression was adopted to explore influencing factors related to mental health problems (e.g., symptoms of depression and anxiety). Variables with significant differences in the bivariate analysis were included in the multiple logistic regression analysis. All of the data analysis was performed through the SPSS 22. The threshold for statistical significance was set at 2-sided *p* < 0.05.

## 3 Results

### 3.1 Demographic information

Out of the 90,118 adolescent and youth students from 162 schools in Sichuan Province, China, a total of 82,873 (92.0%) participants completed the questionnaires, 7,245 (8.0%) declined to participate in the survey. Table 1 shows the demographic and other information of the participants. Of 82,873 participants, 47,487 (57.3%) participants were female, 20,471 (24.7%) participants were the only child at home, 73537 (88.7%) participants were Han Chinese, 36,111 (43.6%) participants were high school students, and 24,157 (29.1%) participants were middle school students. 33,314 (40.2%) participants were confirmed or suspected infection of COVID-19.

**Table 1:** Demographic information of participants (*N* = 82,873).

	Total	Group 1	Group 2	Group 3	Group 4	*p*
	*N* (%)	*N* (%)	*N* (%)	*N* (%)	*N* (%)	
Overall	82,873 (100)	492	896	54,111	27,374	
Age						<0.001
< 18	57,208 (69.0)	433 (88.0)	742 (82.8)	47,015 (86.9)	9,018 (32.9)	
≥18	25,665 (31.0)	59 (12.0)	154 (17.2)	7,096 (13.1)	18,356 (67.1)	
Grade						<0.001
Middle school	24,157 (29.1)	197 (40.0)	296 (33.0)	17,941 (33.2)	5,723 (20.9)	
High school	36,111 (43.6)	252 (51.2)	477 (53.2)	30,706 (56.7)	4,676 (17.1)	
College and university	22,605 (27.3)	43 (8.7)	123 (13.7)	5,464 (10.1)	16,975 (62.0)	
Sex						<0.001
Male	35,386 (42.7)	232 (47.2)	424 (47.3)	23,291 (43.0)	11,439 (41.8)	
Female	47,487 (57.3)	260 (52.8)	472 (52.7)	30,820 (57.0)	15,935 (58.2)	
Sex orientation						<0.001
Homosexual orientation	1,178 (1.4)	4 (0.8)	5 (0.6)	285 (0.5)	884 (3.2)	
Heterosexual orientation	81,695 (98.6)	488 (99.2)	891 (99.4)	53,826 (99.5)	26,490 (96.8)	
Ethnicity						<0.001
Han	73,537 (88.7)	433 (88.0)	791 (88.3)	48,793 (90.2)	23,520 (85.9)	
Other ethnic group	9,336 (11.3)	59 (12.0)	105 (11.7)	5,318 (9.8)	3,854 (14.1)	
Monthly family income, ¥						<0.001
≤ 4,999	48,451 (58.5)	300 (61.0)	507 (56.6)	30,547 (56.5)	17,097 (62.5)	
5,000–19,999	30,699 (37.0)	157 (31.9)	321 (35.8)	21,006 (38.8)	9,215 (33.7)	
≥20,000	3,723 (4.5)	35 (7.1)	68 (7.6)	2,558 (4.7)	1,062 (3.9)	
Single child	20,471 (24.7)	132 (26.8)	281 (31.4)	13,220 (24.4)	6,838 (25.0)	<0.001
Infected with COVID-19						<0.001
Confirmed or suspected cases	33,314 (40.2)	155 (31.5)	370 (41.3)	23,347 (43.1)	9,442 (34.5)	
Family members infected with COVID-19						<0.001
Confirmed or suspected cases	36,402 (43.9)	179 (36.4)	408 (45.5)	25,427 (47.0)	10,388 (37.9)	
Higher level of psychological stress in the three phases	21,564 (26.0)	124 (25.2)	253 (28.2)	13,138 (24.3)	8,049 (29.4)	<0.001
Parenting style						<0.001
Authoritative parenting (Democratic)	45,120 (54.4)	256 (52.0)	471 (52.6)	30,350 (56.1)	14,043 (51.3)	
Authoritarian parenting (Disciplinarian)	18,593 (22.4)	110 (22.4)	213 (23.8)	12,286 (22.7)	5,984 (21.9)	
Neglectful parenting (Uninvolved)	6,378 (7.7)	47 (9.6)	75 (8.4)	3,741 (6.9)	2,515 (9.2)	
Permissive parenting (Indulgent)	12,782 (15.4)	79 (16.1)	137 (15.3)	7,734 (14.3)	4,832 (17.7)	
Quarantine						<0.001
Not quarantined	59,638 (72.0)	378 (76.8)	632 (70.5)	39,738 (73.4)	18,890 (69.0)	
Quarantine at home	16,881 (20.4)	88 (17.9)	208 (23.2)	10,728 (19.8)	5,857 (21.4)	
Quarantine in designated facilities	4,584 (5.5)	14 (2.8)	33 (3.7)	2,649 (4.9)	1888 (6.9)	
Quarantine in hospitals	505 (0.6)	4 (0.8)	15 (1.7)	269 (0.5)	217 (0.8)	
Others	1,265 (1.5)	8 (1.6)	8 (0.9)	727 (1.3)	522 (1.9)	
Daily life routine						<0.001
Partly or seriously influenced	46,980 (56.7)	299 (60.8)	480 (53.6)	31,119 (57.5)	15,082 (55.1)	
Exercising (nearly a year)	43,819 (52.9)	243 (49.4)	478 (53.3)	29,416 (53.9)	13,952 (51.0)	<0.001
Willingness related to vaccine (during COVID-19)	77,520 (93.5)	305 (62.0)	800 (89.3)	50,717 (93.7)	25,698 (93.9)	<0.001
Willingness related to vaccine (restriction lifted)	68,324 (82.4)	325 (66.1)	642 (71.7)	45,196 (83.5)	22,161 (81.0)	<0.001
Being frontline volunteers	4,173 (5.0)	20 (4.1)	32 (3.6)	1833 (3.4)	2,288 (8.4)	<0.001
Family history of mental disorders	837 (1.0)	4 (0.8)	8 (0.9)	600 (1.1)	225 (0.8)	0.002

G1, Participants who did not receive the COVID-19 vaccine; G2, Participants who received one dose of vaccine; G3, Participants who received two doses of vaccine; G4, Participants who received three or more doses of vaccine.

### 3.2 The mental health problems and behaviors among four groups

**Figure 1:**
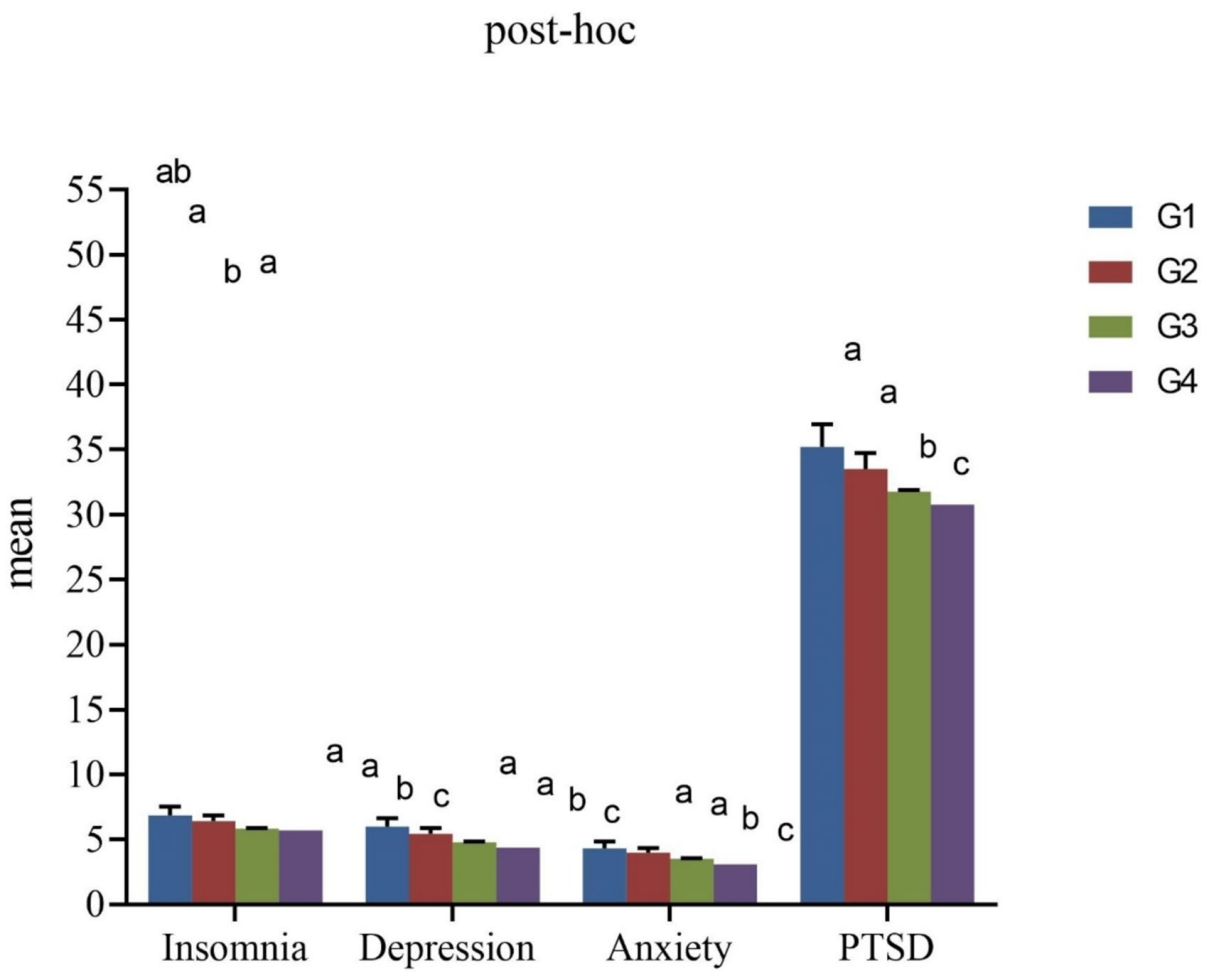
The mean comparison of mental health problems among 4 groups. G1, Participants who did not receive the COVID-19 vaccine; G2, Participants who received one dose of vaccine; G3, Participants who received two doses of vaccine; G4, Participants who received three or more doses of vaccine. Marking as the same letter indicates no significant difference.

Table 2 and Figure 1 show the differences in mental health problems and behaviors among the four groups. The rates of depression symptoms, anxiety symptoms, PTSD, insomnia, and lifetime suicidal ideation were 38.1, 31.8, 33.9, 34.0, and 25.7%, respectively. For depression, anxiety, PTSD, and insomnia, there were significant differences between any two groups except for G1 and G2. Moreover, the rates of mental health problems in G1 were significantly higher than that in G2, G3, and G4 (Depression: 44.3, 40.6, 39.1, 35.9%; Anxiety: 37.8, 35.6, 33.0, 29.3%; PTSD: 40.2, 37.9, 34.8, 31.8%; Insomnia: 39.2, 36.6, 34.2, 33.5%) (*p* < 0.05).

**Table 2:** The comparison of mental health problems and behaviors among 4 groups.

	Total	G1	G2	G3	G4	F/χ2	*p*
	*N* (%)	*N* (%)	*N* (%)	*N* (%)	*N* (%)		
Depression (PHQ-9 score: ≥ 5)	31,564 (38.1) [37.6–38.6]	218 (44.3)	364 (40.6)	21,148 (39.1)	9,834 (35.9)	87.553	<0.001
Anxiety (GAD-7 score: ≥ 5)	26,391 (31.8) [31.2–32.4]	186 (37.8)	319 (35.6)	17,866 (33.0)	8,020 (29.3)	129.972	<0.001
PTSD (PCL-5 score: ≥ 33)	28,112 (33.9) [33.4–34.4]	198 (40.2)	340 (37.9)	18,856 (34.8)	8,718 (31.8)	88.442	<0.001
Insomnia (ISI score: ≥ 8)	28,178 (34.0) [75.7–76.3]	193 (39.2)	325 (36.3)	18,496 (34.2)	9,164 (33.5)	12.185	0.007
Lifetime Suicide Ideation	21,292 (25.7)	142 (28.9)	270 (30.1)	14,453 (26.7)	6,427 (23.5)	111.47	<0.001
Substance abuse	2,302 (2.8)	17 (3.5)	24 (2.7)	1,437 (2.7)	824 (3.0)	9.331	0.025
Drinking	8,579 (10.4)	58 (11.8)	101 (11.3)	4,497 (8.3)	3,923 (14.3)	711.906	<0.001
Smoking	3,007 (3.6)	39 (7.9)	71 (7.9)	2,818 (5.2)	2,477 (9.0)	444.428	<0.001
School bullying	19,190 (23.2)	145 (29.5)	256 (28.6)	13,384 (24.7)	5,405 (19.7)	280.540	<0.001
Cyber bullying perpetration	6,742 (8.1)	55 (11.2)	119 (13.3)	4,275 (7.9)	2,293 (8.4)	43.972	<0.001
Cyber bullying victimization	14,637 (17.7)	101 (20.5)	207 (23.1)	9,720 (18.0)	4,609 (16.8)	37.198	<0.001

G1, Participants who did not receive the COVID-19 vaccine; G2, Participants who received one dose of vaccine; G3, Participants who received two doses of vaccine; G4, Participants who received three or more doses of vaccine.

From G1 to G4, the rates of school bullying were decreased gradually (29.5, 28.6, 24.7, 19.7%) (*p* < 0.001). The highest rates of cyberbullying perpetrators and cyberbullying victims were in G2 (13.3, 23.1%), and the lowest rates of cyberbullying perpetrators and cyberbullying victims were in G3 (7.9%) and G4 (16.8%), respectively (*p* < 0.001). The rates of drinking and smoking in G4 (14.3, 9.0%) were significantly higher than that in other three groups (Drinking: 11.8, 11.3, and 8.3%; Smoking: 7.9, 7.9, and 5.2%) (*p* < 0.001). The highest rate of lifetime suicidal ideation was in G2 (30.1%), followed by G1 (28.9%), G3 (26.7%), and G4 (23.5%) (*p* < 0.001).

### 3.3 Influencing factors of mental health problems

**Figure 2:**
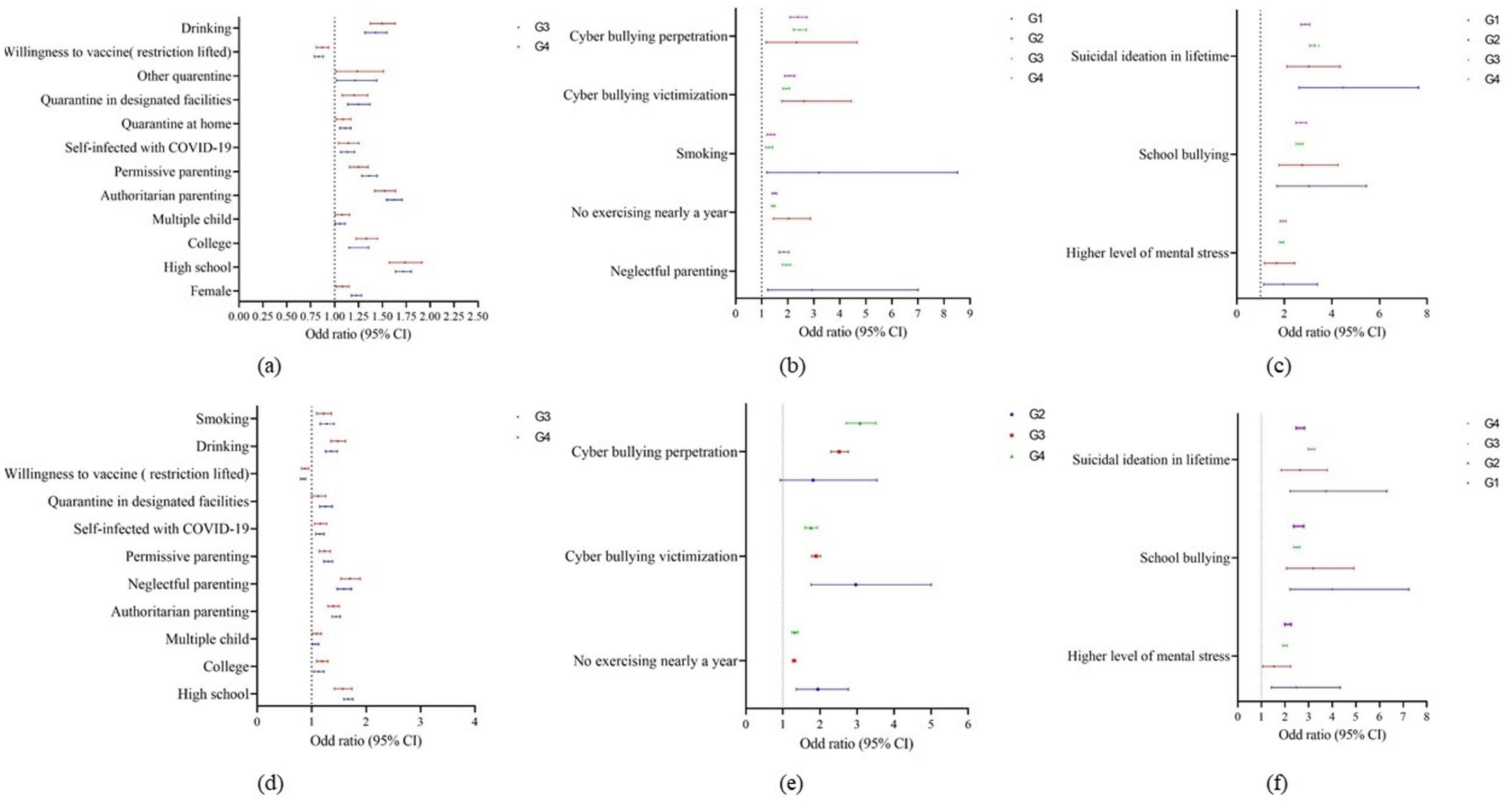
The influence factors of depression and anxiety. G1, Participants who did not receive the COVID-19 vaccine; G2, Participants who received one dose of vaccine; G3, Participants who received two doses of vaccine; G4, Participants who received three or more doses of vaccine; **(A)** The influence factors of depression among 2 groups. **(B)** The influence factors of depression among 3 groups. **(C)** The influence factors of depression among 4 groups. **(D)** The influence factors of anxiety among 2 groups. **(E)** The influence factors of anxiety among 3 groups. **(F)** The influence factors of anxiety among 4 groups.

Figure 2 shows the results of the logistic regression analysis of influencing factors of depression and anxiety. Among participants in G3 and G4, high school (Depression: OR = 1.720, 1.737; Anxiety: OR = 1.673, 1.574; *p* < 0.001), college (Depression: OR = 1.153, 1.335; Anxiety: OR = 1.129, 1.192; *p* < 0.05), being a child in multiple-child-family (Depression: OR = 1.057, 1.080; Anxiety: OR = 1.074, 1.093; *p* < 0.05), being infected with COVID-19 (Depression: OR = 1.135, 1.144; Anxiety: OR = 1.150, 1.162; *p* < 0.05) were risk factors for depression and anxiety. Participants with the willingness for vaccination after the lifting of COVID-19 restrictions in G3 and G4 had significantly lower risk for depression (OR = 0.834, 0.872, *p* < 0.001) and anxiety (OR = 0.843, 0.876, *p* < 0.001) than those without the willingness.

Compared with authoritative parenting style, symptoms of depression and anxiety were also significantly associated with authoritarian parenting style (Depression: OR = 1.625, 1.526; Anxiety: OR = 1.450, 1.400; *p* < 0.001), and permissive parenting style (Depression: OR = 1.363, 1.250; Anxiety: OR = 1.305, 1.238; *p* < 0.001) in G3 and G4. Also, for participants in G3 and G4, drinking (Depression: OR = 1.428, 1.498; Anxiety: OR = 1.361, 1.482, *p* < 0.001) was risk factors for depression and anxiety.

For participants in G2, G3, and G4, the risk of depression and anxiety were significantly higher for both cyberbullying perpetration (Depression: OR = 2.338, 2.460, 2.390; Anxiety: OR = 1.814, 2.520, 3.082) and cyberbullying victimization (Depression: OR = 2.624, 1.937, 2.070; Anxiety: OR = 2.968, 1.892, 1.756). No physical exercise habits in the past year was also a risk factor for depression (OR = 2.036, 1.447, 1.490, *p* < 0.001) and anxiety (OR = 1.940, 1.292, 1.322, *p* < 0.001).

In these four group, suicidal ideation (Depression: OR = 4.474, 3.027, 3.447, 2.872; Anxiety: OR = 3.734, 2.639, 3.119, 2.642; *p* < 0.001), higher level of psychological stress (Depression: OR = 1.958, 1.679, 1.883, 1.946; Anxiety: OR = 2.484, 1.534, 1.995, 2.120; *p* < 0.001) and school bullying (Depression: OR = 3.030, 2.750, 2.622, 2.698; Anxiety: OR = 3.997, 3.191, 2.494, 2.570; *p* < 0.001) were risk factors for depression and anxiety.

## 4 Discussion

This is the first study to explore the vaccination status and mental health issues among adolescent and youth students in Sichuan, China, following the lifting of COVID-19 restrictions. The results show a correlation between COVID-19 vaccination and the protection of mental health among these students (e.g., reducing the incidence of depression and anxiety). This correlation may help explain why the rates of depression symptoms, anxiety symptoms, insomnia, and PTSD have gradually decreased as the number of COVID-19 vaccine doses administered has increased. The majority of participants (99.4%) in this study received at least one dose of the vaccine. Participants with homosexual orientation, drinkers, and smokers were more likely to have received three or more doses of the COVID-19 vaccine. These findings align with previous study indicating that individuals with health issues, such as persons with mental disorders, have a high acceptance rate and willingness to pay for COVID-19 vaccines (31). The results of this study provide evidence for future health policymaking regarding vaccination for different populations.

The results of this study showed that COVID-19 vaccinations had a positive relationship with mental health status in adolescent and youth students. The more doses of vaccines participants received, the less likely they suffered from mental health problems such as depression, anxiety, PTSD and insomnia, which is consistent with previous study (13), but not another study (25). The results suggest that vaccination would not only protect physical health, but also improve mental health in students, in line with prior studies (13, 32). There are possible following reasons that might contribute to the results. First, COVID-19 vaccine protects people from infection, hospitalization, and death, which make people feel less anxious. According to the previous studies, COVID-19-related mental health problems are positively correlated with the number of new cases (33, 34). The COVID-19 vaccine help reduce the severe cases, potentially contributing to a decrease in COVID-19-related mental illnesses. Second, the promotion of COVID-19 vaccine helps with the recovery of economy that might relief people’s anxious about their economic status. Prior study shows that the promotion of COVID-19 vaccine could create jobs and boost GDP (35). Third, vaccination helps lift the COVID-19 restrictions such as social distance and quarantine, and the interaction between family and friends could help reduce anxiety and depression. Prior study shows that people who feel stress about social distance would be more likely to get vaccination (36), suggesting that the psychological stress that brought by COVID-19 restrictions might be reduced by COVID-19 vaccination. Thus, it is important to promote vaccination for those impacted by COVID-19, especially during the COVID-19 pandemic.

This is the first study to explore the correlation between COVID-19 vaccination and school bullying and cyberbulling. The results of this study showed that the more doses of vaccination the students received, the lower percentage of school bullying and mental health problems they suffered from. This result implies a correlation between bullying and negative mental health status, which is consistent with previous studies (37–39). Previous study has shown a significant association between bullying experiences and mental health issues, especially among adolescents (40). The association between bullying experiences and mental health issues may be linked to biological factors such as changes in the neuroendocrine system. Individuals who suffer from bullying often experience high levels of psychological stress (41). And previous study has shown that chronic psychological stress can lead to dysfunction of the hypothalamic–pituitary–adrenal (HPA) axis, which is closely associated with the onset of various mental disorders (42). Also, chronic stress might lead to a reduction in hippocampal volume, which is associated with damage to brain regions involved in memory processing and emotional regulation (43). The lack of social capital might be another possible explanation (44). Social capital, resources that are accessed by individuals as a result of their membership of a network or group, was associated with bullying, as social capital could be a predictor of cyberbullying (45), and social capital could mediate bullying and young people’s subjective well-being (46). On the other hand, mental health problems may also precede bullying, which means that youth students with emotional, behavioral and developmental problems might be more likely to be involved in bullying (47, 48). In this study, unvaccinated students may become victims of bullying because of their psychological or physical status that prevented them from receiving COVID-19 vaccine. As bullying is one of the most modifiable risk factors for mental health problems (49), schools and students’ parents could be important to prevent youth students’ bullying, as well as pay attention to those vulnerable groups that may become victims of bullying.

The results of this study suggest that psychological stress is a risk factor for mental health problems, and vaccination may not reduce the psychological stress of those impacted by COVID-19 pandemic. Evidence showed that the risks of anxiety and depression were associated with individuals’ attitudes toward to vaccines, and individuals who believed the safe and protection of vaccine would have lower rates of depression and anxiety (23). So concerning about the safety of vaccines may be one of the causes of psychological stress for the unvaccinated people, and the causes of psychological stress of the vaccinated population might be varied. Students usually have the pressure of compulsory vaccination (50). Individuals concerning about inadequate vaccination might take more doses of vaccines, and be more sensitive to COVID-19-related problems at the same time. Thus, how to reduce the psychological stress should be important for promotion of vaccination and improving mental health among individuals impacted by COVID-19 pandemic.

### 4.1 Strengths and limitations

To our knowledge, this is the first study to investigate the correlations between vaccination and school bullying, especially after the lifting of COVID-19 restrictions in China. Moreover, this is a study with a large sample size to explore the relationship between doses of COVID-19 vaccines and mental health problems. The results of this study provide unique insights for future vaccine policy-making and psychosocial interventions. Also, the questionnaire of survey was released to students directly through their teachers and professors, which may improve the quality of data collection.

While this study provides an overview of mental health and the doses of vaccine during and after COVID-19 pandemic, this study was conducted only in Sichuan province, China. The results of this study may not generalize to other areas in China. This study is a cross-sectional design, and no causal associations should be inferred. Further long-term follow-up studies should be conducted in this area. Moreover, the mental health scales utilized in this research rely on self-reported data, which may be influenced by social desirability bias, recall bias, biases potentially inherent in educational settings, or the respondent’s emotional state, potentially leading to overestimation or underestimation of mental health levels. Additionally, this study did not fully exclude all possible confounding variables, such as socioeconomic status, educational level, cultural differences, etc. These factors may be associated with mental health status and could potentially influence the interpretation of the research findings. Future research should take these factors into consideration and employ more comprehensive analytical approaches to enhance the generalizability and validity of the results.

## 5 Conclusion

This study aimed to examine the vaccination status of adolescent and youth students and mental health problems and behaviors during and after the COVID-19 pandemic. This study showed that COVID-19 vaccinations had a positive impact on the mental health of adolescent and youth students. The more doses of vaccines the students received, the less likely they suffered from mental health problems such as depression, anxiety, PTSD and insomnia. The results also reveal that different groups of students might have different vaccine acceptance. Participants who were homosexual orientation, drinkers, and smokers were more likely to get three or more doses of vaccines. Moreover, this study suggests a correlation between the doses of COVID-19 vaccines and school bullying and cyberbullying, indicating that the more doses of COVID-19 vaccines the students received, the less school bullying they experienced. Further health policies of vaccination and psychosocial interventions should be developed for promotion of vaccination and mental wellbeing of students.

## Data Availability

The raw data supporting the conclusions of this article will be made available by the authors, without undue reservation.
